# Evaluation of He4 Use in the Diagnosis of Ovarian Cancer: First and Second Recurrence, and an Analysis of HE4 Concentration during Second- and Third-Line Chemotherapy

**DOI:** 10.3390/diagnostics13030452

**Published:** 2023-01-26

**Authors:** Anita Chudecka-Głaz, Aleksandra Strojna, Kaja Michalczyk, Sylwia Wieder-Huszla, Krzysztof Safranow, Edyta Skwirczyńska, Anna Jurczak

**Affiliations:** 1 Department of Gynecological Surgery and Gynecological Oncology of Adults and Adolescents, Pomeranian Medical University, 70-204 Szczecin, Poland; 2 Department of Clinical Nursing, Pomeranian Medical University, 71-210 Szczecin, Poland; 3 Department of Biochemistry and Medical Chemistry, Pomeranian Medical University, 70-111 Szczecin, Poland; 4 Department of History of Medicine and Medical Ethics, Pomeranian Medical University in Szczecin, 70-204 Szczecin, Poland

**Keywords:** ovarian cancer, diagnosis, monitoring, chemotherapy, HE4

## Abstract

HE4 is a commonly used tumor marker for ovarian cancer (OC) diagnosis. In our study, we aimed to assess its use in the diagnosis of subsequent OC recurrences and to evaluate its changes during recurrence diagnosis and the subsequent lines of chemotherapy treatment. This retrospective single center study was conducted on 188 patients treated for ovarian cancer recurrence at the Department of Gynecological Surgery and Gynecological Oncology. The sensitivity and specificity of HE4 for patient survival prediction were analyzed using Receiver Operating Characteristics (ROC) and area under the curve (AUC) with 95% confidence intervals (95% CI). Survival times to reach one of the endpoints (OS, PFS, TFI, PFS2, TFI2) were analyzed using Kaplan–Meier curves. Elevated HE4 levels at the time of first relapse diagnosis, and after the third and the last course of second-line chemotherapy, significantly influences the time from OC diagnosis until first disease recurrence (PFS2) (*p* = 0.005, *p* = 0.015 and *p* = 0.002, respectively). Additionally, elevated serum HE4 concentration at the time of OC diagnosis (*p* = 0.012), and its later recurrence (first (*p* < 0.001), and second recurrent diagnosis (*p* = 0.143)) significantly influences patient OS. Increased HE4 concentration at the end of chemotherapeutic treatment negatively affects overall patient survival ((*p* = 0.006 for second line chemotherapy and (*p* = 0.022) for elevated HE4 concentration after the last course of third-line chemotherapy). Our preliminary results show an encouraging diagnostic and prognostic role of HE4 in recurrent ovarian cancer. HE4 measurements at different treatment time points during the second- and third-line chemotherapy treatment seem to correlate with patient survival.

## 1. Introduction

Ovarian cancer (OC) is a chronic disease with several options for modern cancer treatment, including multiple therapeutic options that combine not only chemotherapy but also PARP inhibitors and anti-angiogenic therapies. Its natural course is characterized by consequent successive periods of remission and relapse, with decreasing durations of disease-free periods. However, OC remains the fifth most lethal malignancy among women and the leading cause of mortality from reproductive organ cancers in developed countries [1,2,3,4]. Due to the high mortality, numerous scientific studies are being conducted to find new methods of treatment that will enable better patient survival. Additionally, new diagnostic, prognostic and predictive biomarkers of high specificity and sensitivity are of high demand to allow early diagnosis of OC and patient stratification.

Several attempts were made to use tumor markers as screening tests [5,6]; however, they have failed to meet the population criteria of screening studies. A recent UK Collaborative Trial of Ovarian Cancer Screening (UKCTOCS) did not show a reduction in mortality in a screening trial using transvaginal ultrasound and/or serum CA125 measurement, illustrating an urgent need for additional ovarian cancer-specific biomarkers [6]. In the diagnostic processes, ultrasound examination is commonly used, and if performed by an experienced expert clinician guarantees the highest sensitivity and specificity in the assessment of the risk of malignancy of ovarian tumors [7]. In 2000, a group of researchers from the International Ovarian Tumor Analysis (IOTA) presented universal definitions to describe ovarian pathologies to standardize the ultrasound examination nomenclature and allow identical interpretation of the ultrasound image by different researchers around the world [8,9]. In order to improve the diagnostic possibilities used in the diagnosis of malignant ovarian lesions, numerous different models and scoring systems have been developed. The Risk of Malignancy Index (MRI) is the most commonly used, which uses the concentration of the CA125 marker, patient menopausal status, and five ultrasound parameters (multiventricular cyst, solid elements, ascites, bilateral tumors, metastases). A milestone, which allowed for better differentiation between benign lesions and ovarian cancer, was the introduction of the Risk of Ovarian Malignancy Algorithm (ROMA), which combines the determination of HE4 and CA125 in one test, accounting for a patient’s menopausal status. A joint determination of HE4 and CA125 has led to a significantly higher accuracy than separate, mono-determination of either CA125 or HE4 [10]. The concentrations of both CA125 and HE4 can be altered due to multiple factors. High CA125 levels are often noticed among patients suffering from benign gynecological conditions including endometriosis, pelvic inflammatory disease and benign ovarian tumors. Increased HE4 levels were found among patients diagnosed with mesothelioma, lung/breast adenocarcinoma and endometrial cancer, or renal failure; however, this was not found in benign pelvic diseases [11,12,13].

Despite aggressive combined treatment, including chemotherapeutical and surgical treatment, approximately 75–85% of ovarian cancer patients relapse after first-line treatment. Recurrent ovarian cancer is a chronic and incurable disease. In FIGO stage I, recurrences are rarely observed (10%). However, they become more frequent among patients initially diagnosed at higher stages, as among stage II patients approximately 30% of patients will face a recurrence, and 85% of patients who were diagnosed at stages III and IV will face a recurrence [14]. The time from the end of primary treatment to the appearance of the first symptoms of relapse (either an increase in marker concentration and/or the appearance of clinical symptoms) may vary in length and range from several weeks to several years [15]. The median onset time of recurrence for patients initially diagnosed with stage IIB-IV is approximately 22 months [16]. According to current knowledge, the first measurable sign of recurrence is an increase in CA125 concentration, which usually precedes clinical symptoms of the disease by an average of 5 months [17,18]. Until recently, CA125 measurement was the only marker used to monitor the treatment of ovarian cancer patients. According to the National Comprehensive Cancer Network (NCCN) guidelines, a clinical trial with the determination of the CA125 marker is recommended for patients completing the first-line treatment [19,20]. The sensitivity of CA125 in the detection of the recurrence of ovarian cancer was found to equal 83.9% [21].

HE4 (epididymal epithelial cell protein 4) is a member of the whey acidic protein four-disulfur core family. It acts as a protease inhibitor, inhibiting serine, aspartyl and cysteine proteases; however, its exact function remains unknown [22]. In humans, it is encoded on chromosome 20q13. Amplification of this region has been documented in breast and ovarian cancer [23,24]. Moreover, HE4 was found to act as an inhibitor of trypsin degradation, thus retaining its activity. As previous studies have shown trypsin to play a role in tumorigenesis and cancer progression [25], its increased levels may support the hypothesis that trypsin has a tumorigenic role in OC, which can be mediated by PAR2 (protease activated receptor 2) receptor and further potentiated by HE4 [26,27,28].

For several years, HE4 has been found to play an important role in the diagnosis of ovarian cancer, especially using the ROMA algorithm. It was first identified by researchers at the Pacific Northwestern Research Institute in Seattle, and the first report on the possible use of HE4 as a tumor marker in ovarian cancer was published by Hellstromi et al. in 2003 [29]. The HE4 expression in ovarian tumors depends on the histological subtype. It is expressed in almost all serous and endometrial ovarian neoplasms, which constitute the majority of ovarian cancers [30]. In contrast, mucinous epithelial tumors and some germinal tumors rarely express HE4 [31]. Some studies suggest increased serum HE4 expression in nearly 92% of patients with ovarian cancer, showing similar sensitivity and increased specificity to the CA125 marker [32]. Previous studies have also investigated the possible predictive value of both CA125 and HE4 in ovarian cancer recurrence. Steffensen et al. [33] found CA125 not to be a significant prognostic factor for PFS, while HE4 was found to act as a sensitive marker of OC recurrence during the first 6 months of patient follow-up.

The aim of the study was to assess the use of HE4 in the diagnosis of subsequent recurrences of ovarian cancer and to evaluate its changes during the subsequent lines of chemotherapy.

## 2. Materials and Methods

### 2.1. Patient Characteristics

This single-center study included 188 patients treated for ovarian cancer at the Department of Gynecological Surgery and Gynecological Oncology of Adults and Adolescents, Pomeranian Medical University in Szczecin, Poland from November 2011 to March 2018. The study inclusion criteria were as follows: >18 years of age, surgical treatment for ovarian cancer (either primary or interval cytoreductive surgery), adjuvant or neoadjuvant chemotherapy treatment, including 6 cycles of chemotherapy based on paclitaxel and carboplatin. All first-line chemotherapy patients achieved an objective response to treatment (ORR) confirmed by RECIST 1.1 criteria in computed tomography. Patients with disease progression or no objective response to treatment in CT imaging studies were not included in the study. Patients with incomplete/missing data, patients who did not finish first-line chemotherapy or were lost in follow-up were excluded from the study. The study was retrospective and included data regarding patients’ treatments and 5-year follow-up observations. Clinical data (age, tumor stage according to FIGO, grading, histopathological examination results, tumor marker values and follow-up data were obtained from the archives of the medical records of the Department of Surgical Gynecology and Gynecological Oncology of Adults and Girls of the Pomeranian Medical University and the documentation of the Clinical Outpatient Clinic. All of the histopathological analyses were performed at the Department of Pathomorphology, Pomeranian Medical University.

The final research material consisted of 188 patients aged 18 to 87. For the study purpose, patients were divided into two groups:

- The study group of patients who were diagnosed with a recurrence—96 patients with at least one recurrence, of whom 40 developed a second recurrence during the study period.
- The control group of patients without recurrence—in 92 patients the recurrence was not diagnosed within the observation period.

Patients were classified into platinum-sensitive and platinum-resistant subgroups in accordance to the Gynecologic Cancer Intergroup (GCIG) consensus [34]. Platinum resistance was defined by less than 6 months of remission following chemotherapy, while patients who have initially responded to platinum-based therapy and had a therapy-free interval for over 6 months were considered platinum-sensitive.

Patient characteristics were presented in Table 1.

### 2.2. HE4 Immunoenzymatic Analysis

Serum HE4 levels were analyzed at the time of patient diagnosis, during chemotherapy treatment, after its completion and during the follow-up period. Before each sampling, patients were advised not to take any biotin (vitamin B7) supplements as it might have influenced serum HE4 analysis. Laboratory determinations were conducted at the Department of Laboratory Diagnostics SPSK2 in Szczecin, Poland. Roche enzyme immunoassays (Cobas e) were used for determinations. The reference values for HE4 were set at 70.0 pmol/L, respectively. The concentration of HE4 was determined using an “ECLIA” Elecsys electrochemiluminescence method, using the Roche Cobas E 601 analyzer. The range of the measurement was from 15.0 to 1500 pmol/L. Samples with an HE4 concentration above the measuring range were diluted with a Diluent MultiAssay. The determination was performed in accordance to the manufacturers’ protocol. Values below 70 pmol/L were adopted as the limit value of HE4 concentration.

### 2.3. Statistical Analysis

Mean, standard deviation, median and range were calculated as basic descriptive statistics for the measurable variables. The distribution of the study population was characterized using the Shapiro–Wilk test, which showed that the distribution of the most of the variables significantly differed from the normal distribution. The groups were analyzed using the non-parametric U Mann–Whitney tests. The sensitivity and specificity of HE4 for patient survival prediction were analyzed using the Receiver Operating Characteristics (ROC) method, the area under the curve (AUC) with 95% confidence intervals (95% CI) and statistical significance of the predictive value in comparison to the test with zero predictive value (AUC = 0.5). Survival times to reach one of the endpoints (OS, PFS, TFI, PFS2, TFI2) were analyzed using Kaplan–Meier curves which were compared between groups using the log-rank test. A *p*-value of <0.05 was adopted as the threshold of statistical significance for all analyzed subgroups. Statistical analyses were performed using Statistica 13 program with the “Plus” bundle.

## 3. Results

### 3.1. Comparison of Serum HE4 Levels between Patients with Recurrent Ovarian Cancer and Those Who Were Not Diagnosed with OC Recurrence during the Study Follow-Up

Significantly higher values of HE4 were found at the time of ovarian cancer diagnosis in the group of patients who later recurred from the neoplastic process (648.1 vs. 295.5 pmol/L, *p* = 0.015). Additionally, among the patients who developed OC recurrence during the study period, higher HE4 values were found after the cytoreductive surgery (129.1 vs. 87.8 pmol/L, *p* = 0.009). Statistically significant higher values of HE4 were demonstrated after the third chemotherapy of the first line of treatment (110.3 vs. 77.3 pmol/L, *p* = 0.000). The trend was similar at the end of the first-line chemotherapy treatment. Significantly higher values of HE4 were found among patients who experienced OC relapse (95.2 vs. 79.2 pmol/L, *p* = 0.294). The results are demonstrated in Table 2.

### 3.2. Assessment of Serum HE4 Concentrations at Various Study Checkpoints during the Second and Third Line Chemotherapy, Accounting for OC Prognostic Factors

#### 3.2.1. Serum HE4 Assessment at the Time of Recurrence Diagnosis, after the Third Course, and after the Last Chemotherapy of the Second Line of Treatment, Accounting for OC Prognostic Factors

We found statistically significant higher HE4 levels at the time of diagnosis of the first recurrence of OC among patients with larger residual tumors after primary surgery >10 mm vs. <10 mm, 293.5 vs. 156.6, respectively (*p* = 0.004). Similar correlations were found after the third (*p* = 0.006) and last chemotherapy of the second line of treatment (*p* = 0.226). Significantly higher values of the HE4 marker were also found in platinum-resistant patients, when compared to platinum-sensitive patients, at the diagnosis of the first relapse (*p* = 0.024), at the third cycle of chemotherapy (*p* = 0.000) and after the completion of the second-line treatment (*p* = 0.000). The specific results are listed in Table 3.

#### 3.2.2. Assessment of HE4 Concentrations at The diagnosis of the Second Recurrence, after the Third Course, and after the Last Chemotherapy of the Third Line of Treatment

At the diagnosis of second recurrence of ovarian cancer, significantly higher HE4 levels were found among patients with left neoplastic residues > 10 mm, when compared to the group without any tumor residues after the primary surgery (*p* = 0.045). In addition, HE4 was significantly higher at the time of second recurrence diagnosis in patients with neoplastic residues > 10 mm vs. > 0 ≤ 10 mm after the primary surgery (*p* = 0.012), Table 4.

### 3.3. Assessment of HE4 Prognostic Value

#### 3.3.1. Assessment of the Prognostic Value of Serum HE4 Values Measured at OC Diagnosis, Predicting OC Recurrence

**Figure 1:**
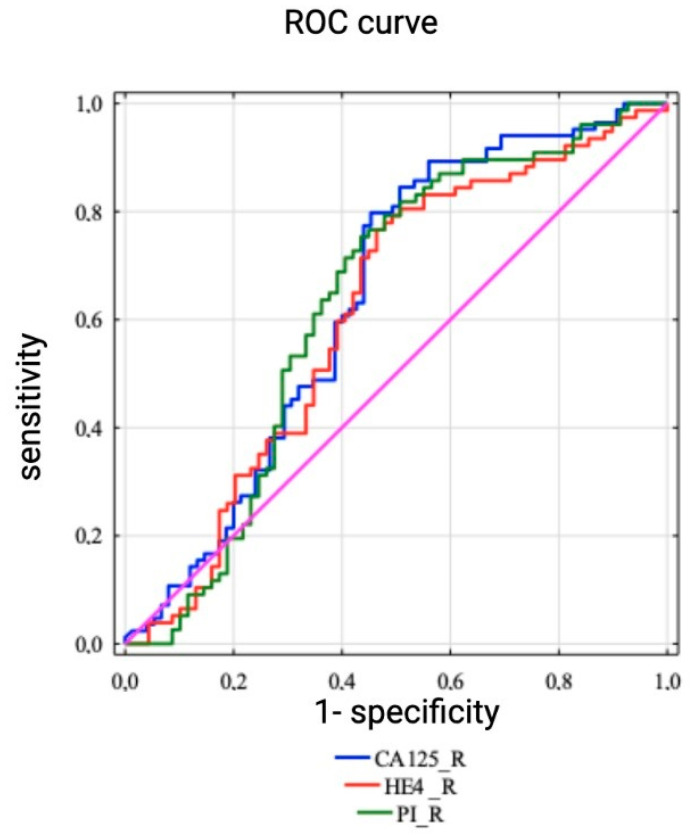
ROC curves to assess the risk of recurrence in the whole population using the HE4 marker.

HE4 specificity and sensitivity analysis showed the area under the ROC curve (AUC) to be 0.61 (95% CI 0.52–0.71), significantly differentiating patients with relapses from patients who did not relapse among the entire population (*p* = 0.017), Figure 1.

#### 3.3.2. Assessment of HE4 Prognostic Values at Different Study Timepoints

The detailed results regarding the prognostic value of HE4 in terms of 2-year and 5-year survival prediction depending on the treatment method and platinum sensitivity are presented in Table A1, Table A2, Table A3, Table A4, Table A5 and Table A6 (Appendix A).

## 4. Assessment of Serum HE4 Concentration and Its Association with the Duration of PFS2 and OS

### 4.1. Assessment of Serum HE4 Concentrations during the Patients’ Follow-Ups and Their Correlation with Overall Survival (OS)

**Figure A1:**
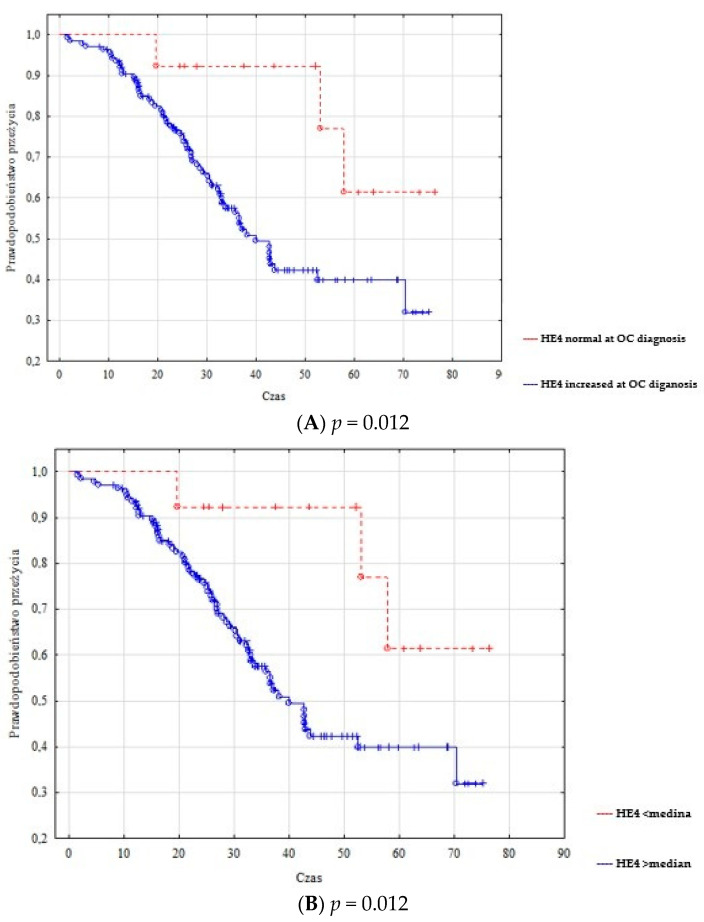

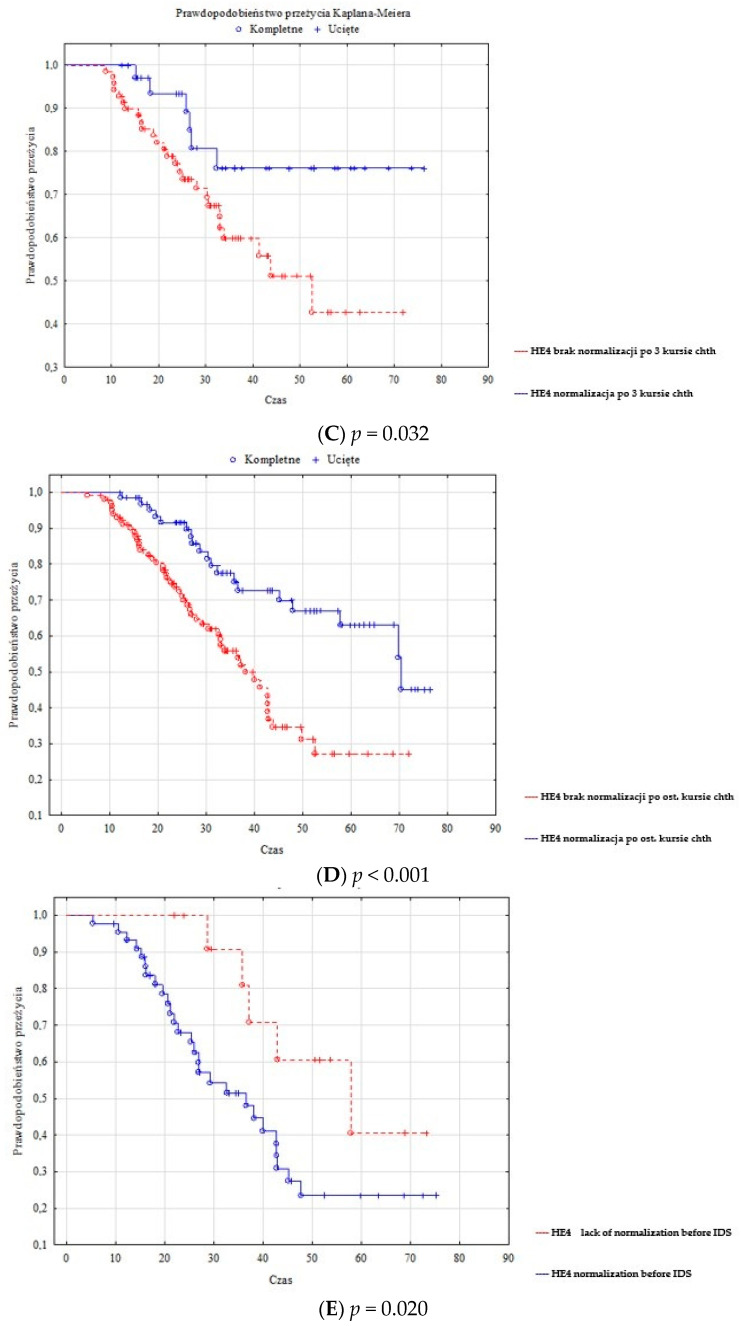

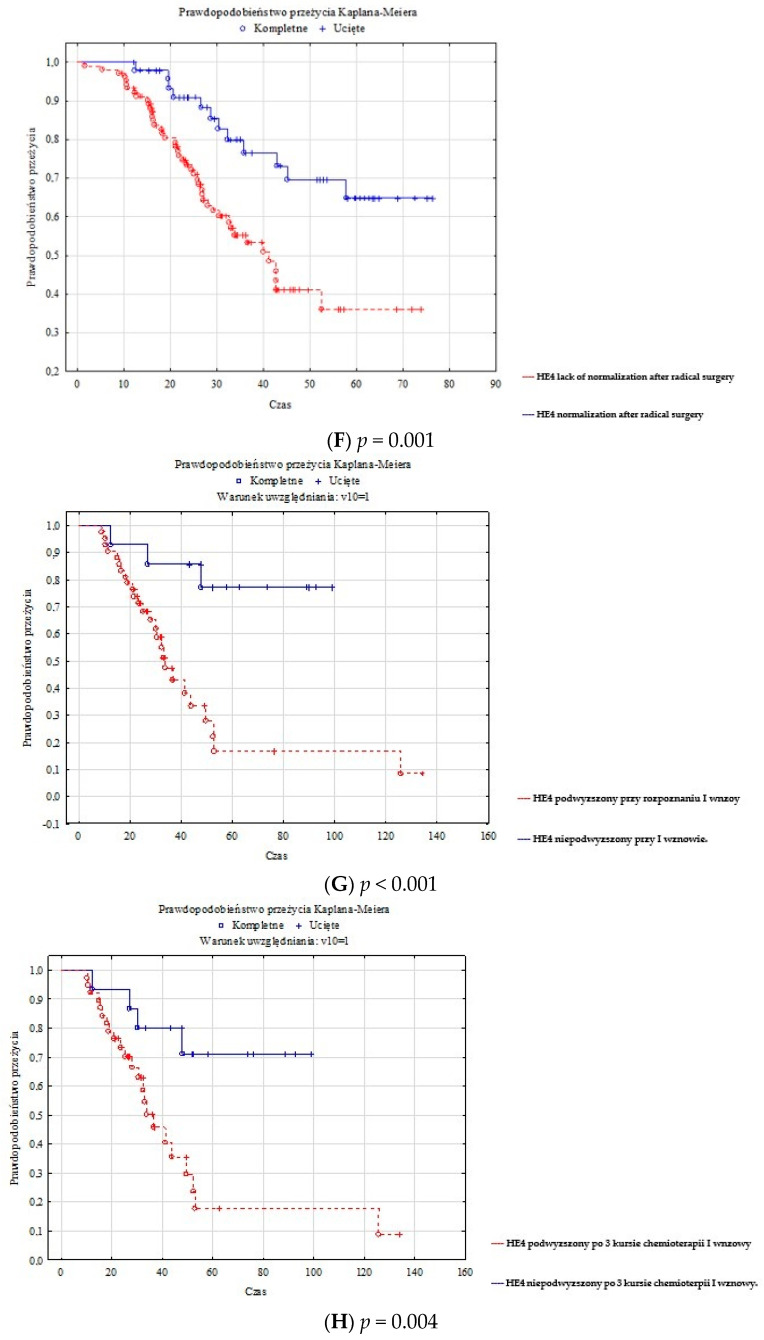

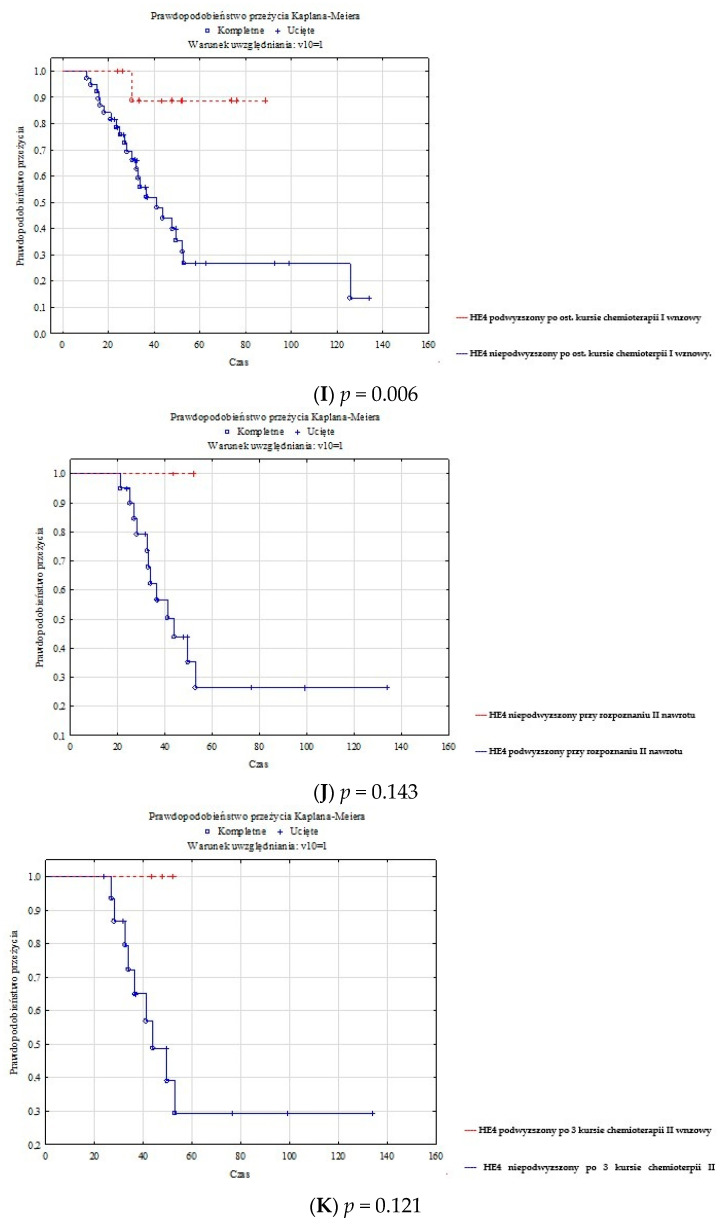

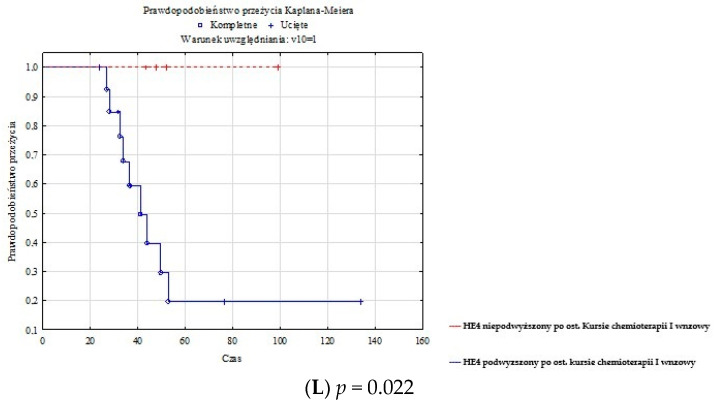
Kaplan–Meier survival curves for overall survival (OS) according to HE4 concentration. (**A**) Elevated serum HE4 concentration at the time of OC diagnosis; (**B**) HE4 > median at the time of OC diagnosis; (**C**) normalization of HE4 values after the third course of first-line chemotherapy; (**D**) normalization of HE4 values after the last course of first-line chemotherapy; (**E**) HE4 normalization prior to IDS; (**F**) HE4 normalization after radical surgery; (**G**) increased HE4 concentration at the diagnosis of first OC recurrence; (**H**) increased HE4 after the third course of second-line chemotherapy; (**I**) increased HE4 concentration after the last course of second-line chemotherapy; (**J**) increased HE4 concentration at second recurrence diagnosis; (**K**) increased HE4 concentration after the third course of third-line chemotherapy; (**L**) increased HE4 concentration after the last course of third-line chemotherapy.

As this part of the study, we have conducted multiple statistical model analyses to analyze the influence of HE4 concentration and its changes at different OC treatment timepoints. Increased serum HE4 concentration at the time of OC diagnosis, HE4 values above median at the time of OC diagnosis, normalization of HE4 levels after the third and last cycle of first line chemotherapy as well as normalization of HE4 levels after either IDS or PDS were found to influence patient OS. The detailed results are presented in Figure A1 (Appendix B).

### 4.2. Serum HE4 during the Follow-Up Period of Patients Diagnosed with OC Recurrence and Its Influence on Progression-Free Survival 2 (PFS2)

**Figure A2:**
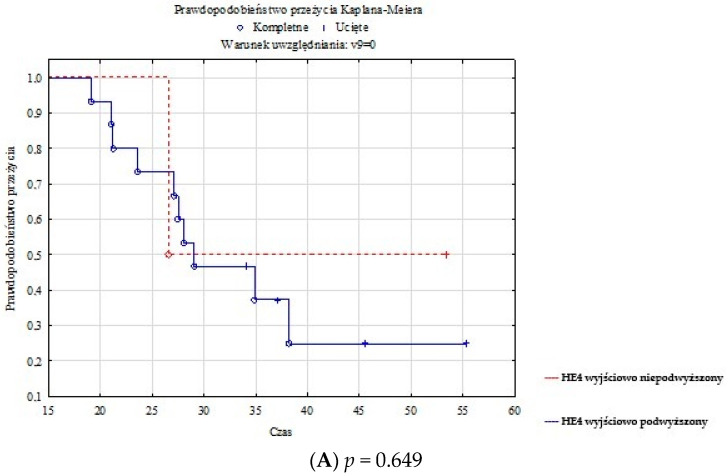

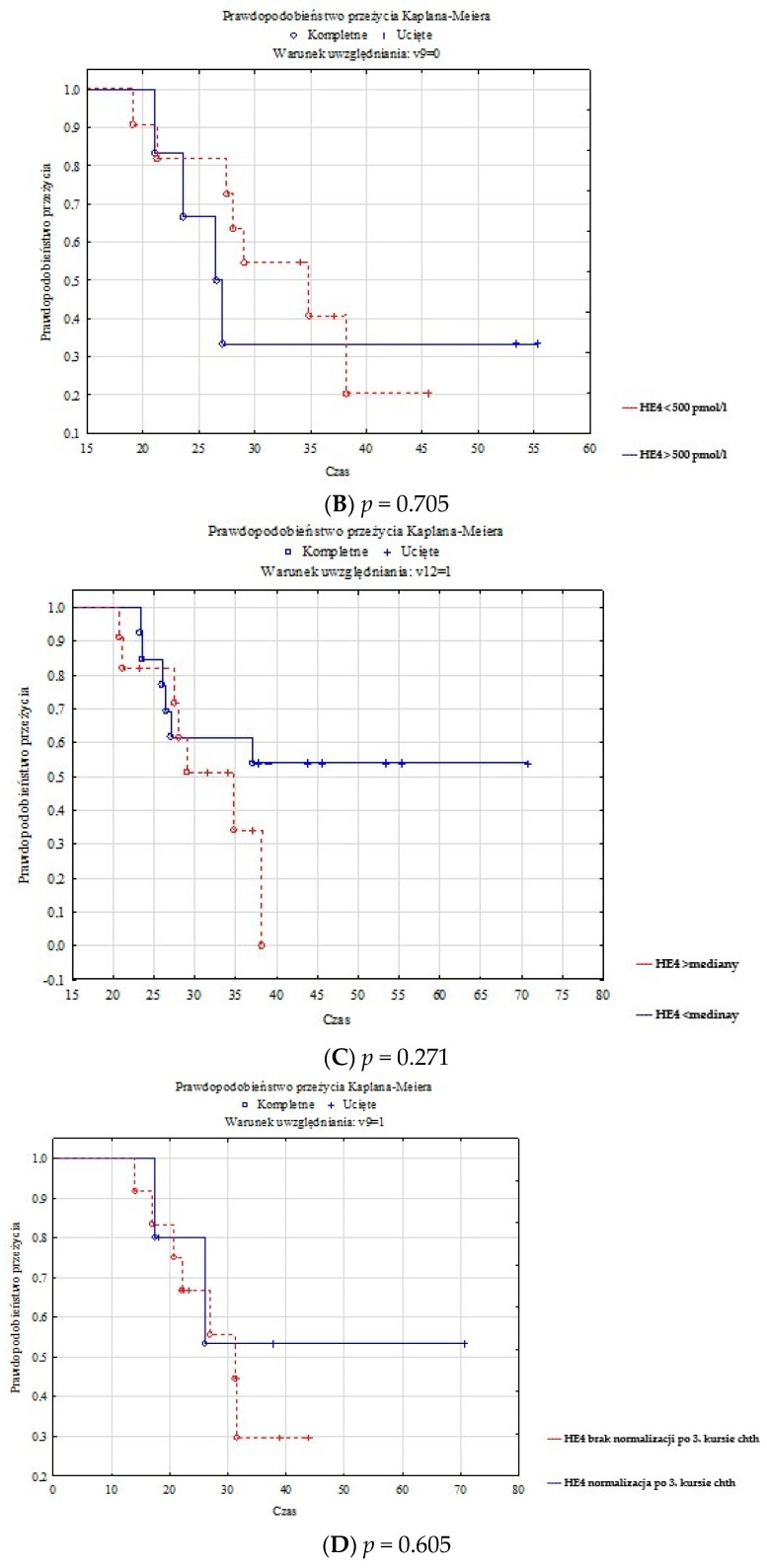

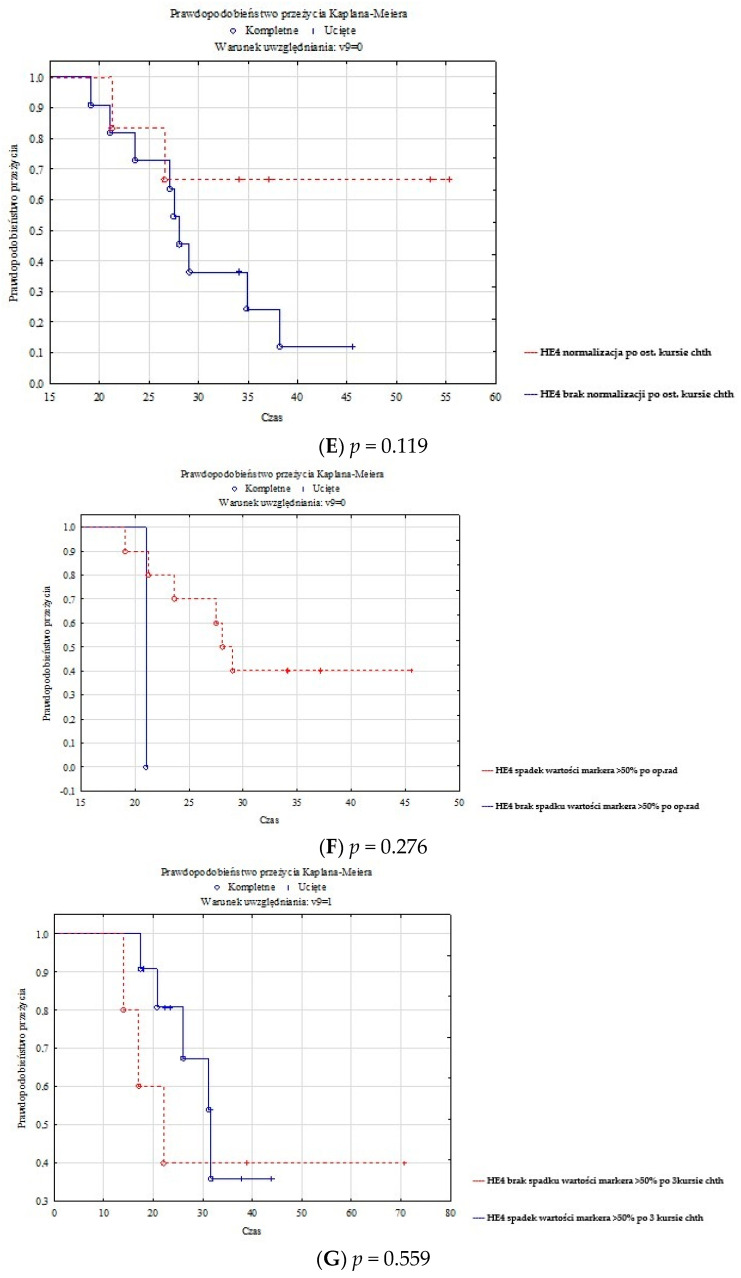

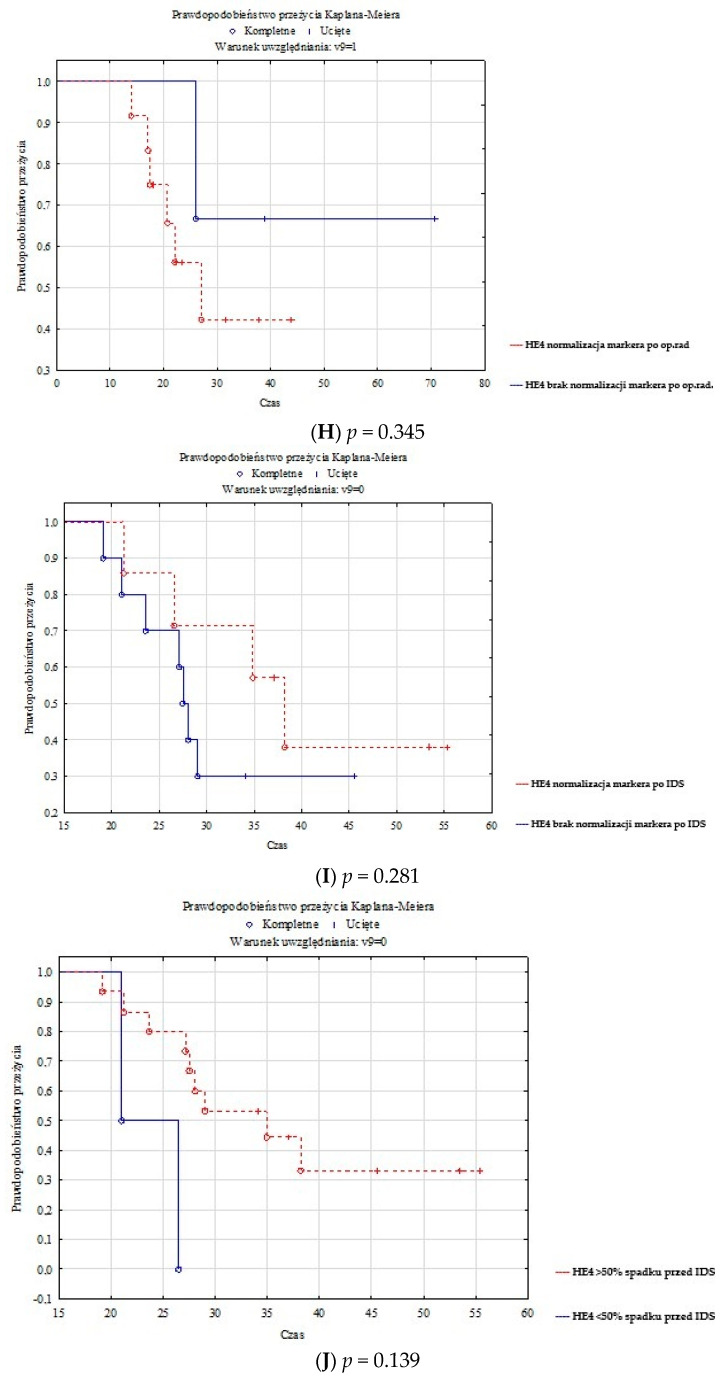

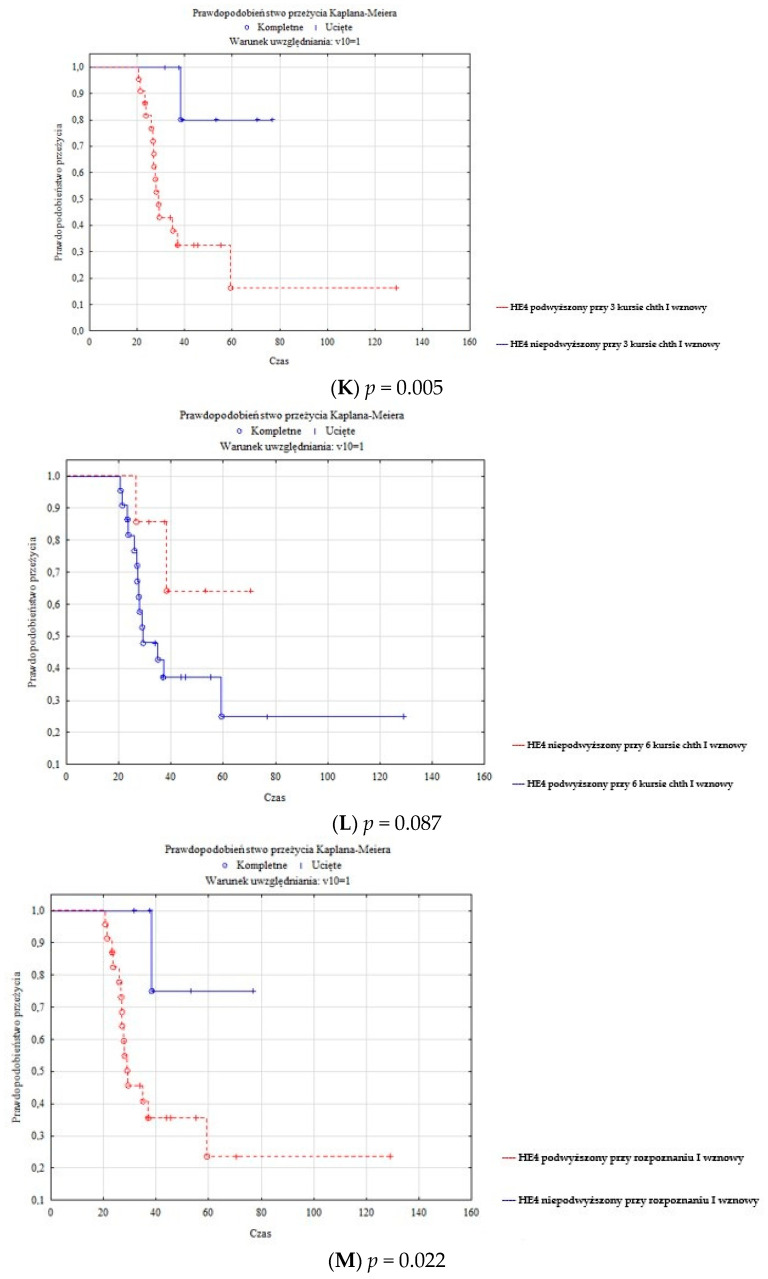
Kaplan–Meier survival curves for progression-free survival 2 (PFS2), the time from disease diagnosis to diagnosis of 2 relapses, according to HE4 concentration. (**A**) Elevated serum HE4 concentration at the time of OC diagnosis; (**B**) HE4 above 500.0 pmol/L; (**C**) HE4 > median at the time of OC diagnosis; (**D**) normalization of HE4 values after the third course of first-line chemotherapy; (**E**) normalization of HE4 values after the last course of first-line chemotherapy; (**F**) HE4 decrease >50% after PDS; (**G**) HE4 decrease >50% after the third course of first-line chemotherapy; (**H**) HE4 normalization after PDS; (**I**) HE4 normalization before IDS; (**J**) decrease of 50% in HE4 concentration before IDS; (**K**) increased HE4 at first OC recurrence diagnosis; (**L**) increased HE4 after the third course of second-line chemotherapy; (**M**) increased HE4 after the last course of second-line chemotherapy.

Among the patients treated with the adjuvant chemotherapy regimen, the PFS2 time, which is the time from disease diagnosis to diagnosis of two relapses, was found to be statistically significantly dependent on HE4 concentration at different treatment timepoints including the time of recurrence diagnosis, and the times of the third and sixth cycles of second-line chemotherapy treatment. In patients who underwent neoadjuvant chemotherapy before IDS, a significant influence of increased HE4 values on PFS was found at the recurrence diagnosis as well as at the time of third cycle of second-line chemotherapy. The specific results are presented in Figure A2 (Appendix C).

## 5. Discussion

To date, CA125 has been the most commonly used and extensively studied tumor marker in the diagnosis and treatment monitoring of ovarian cancer. HE4 is an important and promising tool with diagnostic, prognostic and predictive potential. Even though the role of HE4 has been investigated over the last decades, still, there is little available research confirming its use in OC patients during the follow-up period. In 2009, the US Food and Drug Administration (FDA) agreed to use HE4 to diagnose and monitor ovarian cancer; later in 2011, it has also approved its use in combination with the marker CA125 as a part of the ROMA for the diagnosis of ovarian cancer [35]. In accordance to the recent literature, HE4 has a higher specificity than the most frequently used and widely used marker CA125, while maintaining very high sensitivity [35,36,37]. Unlike Ca125, it is not increased during benign gynecological conditions such as endometriosis, adenomyosis, uterine fibroids or even menstruation, and thus these do not compromise its specificity. However, HE4 concentration can be influenced by altered kidney function and smoking [38]. HE4 was also shown to have a higher sensitivity of Stage I tumor detection, when compared to Ca125 [35].

A meta-analysis by Olsen et al. has shown HE4 to have a higher specificity (0.84 vs. 0.57) and similar sensitivity (0.79 vs. 0.81) to the CA125 marker malignant lesion identification. Similar results were confirmed in different studies [39,40,41]. So far, most of the studies evaluating the role of HE4 in ovarian cancer treatment monitoring have included a limited number of patients. Additionally, the majority have focused on treatment monitoring only during the first line of treatment. Our research is one of a few in the field that has evaluated the changes of HE4 concentration during subsequent lines of chemotherapy and recurrence diagnosis.

The choice of treatment for ovarian cancer recurrence and the response to chemotherapy in the subsequent lines of treatment are primarily influenced by platinum sensitivity, defined by the duration of time from the end of the first-line treatment with platinum compounds to the first or subsequent disease recurrence. As platinum sensitivity is one of the most important OC prognostic factors, in our study, we wanted to evaluate if HE4 correlates with platinum sensitivity and also acts as a prognostic factor at different time points of patient follow-up. Little research has investigated the role of the HE4 marker in predicting platinum sensitivity. Pelissier et al. [42] demonstrated HE4 as a potential tool for platinum sensitivity prediction, showing the HE4 value at 115 pmol/L after the third cycle of chemotherapy to be the best cut-off point for the identification of platinum-sensitive patients. In addition, Angioli et al. [43] showed a possible correlation between platinum sensitivity and HE4 levels. In the group of patients that were diagnosed with a platinum-resistant recurrence during the study period, after the third cycle of first-line chemotherapy, the level of the HE4 marker exceeded 70 pmol/L in all patients (sensitivity 100%, specificity 85%). The reduction of the HE4 marker by 47% after the third cycle of chemotherapy had 83% sensitivity and 87% specificity (PPP 0.86 m NPV 0.85) in predicting platinum sensitivity. Contrary results were presented by Kayser et al. [44], who concluded that the value of the HE4 marker before OC surgery is not an independent prognostic factor for PFS and DFS. However, a different study by Nassir et al. [45] reported the combination of increased CA125 and HE4 markers to negatively influence PFS as it significantly worsened the median PFS (HR 8.14; 95% CI 3.75–17.68, *p* < 0.001) and only slightly worsened PFS in patients that only presented with increased HE4 levels (HR 1.46, 95% CI 0.72–2.96, *p* = 0.292).

In our study, we found significantly higher HE4 levels in platinum-resistant patients compared to the group of platinum-sensitive patients at different study timepoints including the diagnosis of first OC recurrence (*p* = 0.024), third cycle of second-line chemotherapy (*p* = 0.024) and after its completion (*p* < 0.001). However, no statistically significant differences were found at the time of the second OC recurrence or during the third-line chemotherapy (Table 3 and Table 4). We have also analyzed the prognostic values of HE4 in terms of 2-year and 5-year survival predictions. The analysis was conducted separately for platinum-sensitive and platinum-insensitive (platinum-resistant) patients during the second and third line of chemotherapy treatment. Significant prognostic values were more often observed among platinum-sensitive patients when compared to the group of platinum-resistant patients. The amount of statistically significant results was inversely correlated with the duration of therapy, as significant results were less frequent with an increasing line of chemotherapy treatments used (the results regarding the prognostic value of HE4 in terms of 2-year and 5-year survival prediction at the time of second and third line chemotherapy treatment are demonstrated in Table A1, Table A2, Table A3, Table A4, Table A5 and Table A6).

Another prognostic factor that greatly influences ovarian cancer patient survival is complete cytoreduction. Studies show that presence of residual disease after debulking surgery significantly worsens patients prognosis [46,47]. As a part of the study, we have also tried to assess the correlations between serum HE4 and presence of residual disease. In a previous study, Trudel et al. [48] demonstrated a statistically significant relationship between elevated HE4 and the presence of residual disease (*p* < 0.0001). Moreover, in accordance with the results shown by Vallius et al. [49], preoperative serum HE4 assessment may be useful in patient selection for primary cytoreductive surgical treatment, as patients with HE4 > 645 pmol/L were found to be primarily inoperable, regardless of the skills of the surgical team. Similarly, Angioli et al. [43] showed that preoperative HE4 concentration might predict the possibility of optimal cytoreduction, and serve as one of the patient selection criteria for radical surgical treatment.

Chudecka-Głaz [50] et al. assessed the use of various tumor markers, including HE4, in the prediction of optimal cytoreduction. The calculated cut-off value for optimal cytoreduction to R0 of HE4 was established at 218.43 pmol/L with the sensitivity, specificity, PPV and NPV for HE4 equal to 86.6%, 91.3%, 92.9% and 84%, respectively, when compared to CA125 (83.3%, 75%, 80.6%, 78.3%, respectively). Furthermore, Tang et al. [51] investigated the utility of the preoperative value of the HE4 marker in predicting optimal cytoreduction. The cut-off value for HE4 of 473 pmol/L was used. Suboptimal cytoreduction was achieved in 66.7% (38/57) of patients, in whom the HE4 marker value was ≥473 pmol/L, compared to 27.3% (9/33) patients with HE4 < 473 pmol/L. The sensitivity, specificity, PPV and NPV in identifying the population with suboptimal results of cytoreductive surgery were 81%, 56%, 67 and 73%, respectively. As different studies have used different cut-off values, based on the results of performed ROC curve analyzes that measure the effectiveness of diagnostic test, it is difficult to compare study results.

In our research, we found that residual tumor tissue after debulking surgery correlated with higher serum HE4 concentration at the time of first recurrence diagnosis and the duration of second line of chemotherapy treatment in patients with residual tumors >10 mm after the radical surgery, when compared to patients with T < 10 mm (*p* = 0.004). Similar correlations were found before the third chemotherapy and after the last chemotherapy of second-line treatment, with median T > 10 mm 215.4 pmol/L, and T < 10 mm 115.25 pmol/L (*p* = 0.006) before the third cycle of chemotherapy, and 185.9 pmol/L and 101.5 pmol/L (*p* = 0.226) for the last cycle. At the time of second relapse diagnosis, significantly higher median values were observed among patients with residual tumors T > 10 mm than in patients with R0 (*p* = 0.045). A similar trend was noticed in patients with residual tumors T > 10 mm vs. T > 0 ≤ 10 mm (*p* = 0.001). The ROC of serum HE4 for the prediction of residual disease in the control group of patients without disease recurrence during the study period was AUC 0.605 (95% CI 0.435–0.774; *p* = 0.023). The results of the statistical analysis for the study group as well as for the entire population were insignificant.

Several researchers conducted the influence of HE4 on ovarian cancer survival analysis. Peak et al. [52] demonstrated significantly shorter progression-free survival (PFS) among advanced ovarian cancer patients with elevated HE4 at OC diagnosis, compared to patients with non-elevated HE4 levels (20.1 vs. 24.2 months, *p* = 0.029). Similar results were found by Kong et al. [53], who demonstrated HE4 to be an independent prognostic factor for PFS (*p* = 0.036). In addition, Bandiera et al. [54] showed elevated HE4 levels as an independent prognostic factor for shorter OS, DFS and PFS. Decreased OS among patients with high HE4 values was also noticed by Kalapotharakos et al. [55] (HR 2.02, 95% CI 1.1–3.8).

Steffensen et al. [56] showed CA125 and HE4 to be highly predictive for both PFS and OS. His study group has [33] also proven the importance of marker measurement during the follow-up period. Patients, in whom the HE4 value has doubled during the follow-up period either at the third or sixth month of treatment compared to HE4 concentration at the end of the first line of treatment, had a statistically significantly shorter progression-free survival (HR 2.82, *p* = 0.0052 and HR 7.71, *p* < 0.0001, respectively). The multivariate analysis confirmed elevated HE4 concentration at the 6-month follow-up to be a predictor of shorter PFS (HR 8.23, 95% CI, *p* < 0.0001). Another study by Trudel et al. [48] demonstrated increased HE4 > 394 pmol/L is statistically significantly associated with higher mortality (HR = 2.17; 95% CI: 1.42–3.32) and higher risk of neoplastic disease progression (HR = 1.81; 95% CI: 1.21–2.72).

In our research, we have also evaluated the importance of HE4 measurement during the follow-up period. Elevated HE4 levels at the time of first relapse diagnosis, and after the third and the last course of second-line chemotherapy were found to significantly influence the time from OC diagnosis until first disease recurrence (PFS2) (*p* = 0.005, *p* = 0.015 and *p* = 0.002, respectively).

In our study, we have confirmed the role of HE4 as a prognostic OC marker. We found elevated HE4 levels above median at the time of OC diagnosis, its normalization after the third and the last cycle of first-line chemotherapy, and normalization of HE4 values after IDS or PDS to significantly influence patients’ overall survival.

There is a limited number of studies that have evaluated the use of HE4 in OC patient monitoring in patient follow-up after the first-line treatment. In 2012, Schummer et al. [57] compared the use of CA125, HE4, mesothelin and MMP7 in ovarian cancer treatment monitoring. Moreover, the research group have also studied the timing of marker increase before the first clinical symptoms of OC recurrence on a study population of 23 patients. It was observed that the rise of HE4 concentration significantly preceded the rise of other markers, even in patients in whom the CA125 concentration did not elevate before the recurrence diagnosis.

When compared to the previous studies, our study was conducted on a relatively big study sample as almost 200 patients were involved in the final analysis. Our follow-up study has shown that patients, who developed OC recurrence during the study period, had significantly increased serum HE4 levels both at the time of initial OC diagnosis as well as during OC treatment. The median HE4 concentration among patients who developed OC recurrence at the time of primary OC diagnosis was 648.1 pmol/L vs. 295.5 in patients without disease recurrence (*p* = 0.015). Patients, who developed OC recurrence, also demonstrated higher HE4 levels after radical surgery (129.1 vs. 87.8 pmol/L, *p* = 0.009) and after the third cycle of chemotherapy (110.3 vs. 77.3, *p* = 0.000). The HE4 specificity and sensitivity analysis demonstrated the AUC to equal 0.61 (95% CI 0.52–0.71), thus significantly differentiating patients with OC recurrence from patients who did not relapse during the study period (*p* = 0.017).

Additionally, Plotti et al. [58] have studied the role of tumor markers (CA125, HE4 and CA-72.4) in the diagnosis and monitoring of patients with ovarian cancer. He tested markers’ diagnostic potential and then monitored their concentration during the first-line treatment on a limited patient population of 20 patients. The authors found a combination of HE4 and CA72.4 markers to have a higher OC recurrence prediction potential than CA125 alone. In patients with recurrent ovarian cancer, the sensitivity of HE4 was 73.53% and 26.47% for HE4 values > 70 and >150 pmol/L, respectively. Similar results were published by Manganaro et al. [32], who coordinated a retrospective study on a population of 21 patients diagnosed with stage III/IV ovarian cancer after radical surgery and adjuvant chemotherapy. He observed, that among nine patients with cancer recurrence, HE4 concentration was already elevated in 22% of patients 1 to 3 months after the surgery.

Our analysis revealed multiple correlations between serum HE4 concentration and patient 2-year and 5-year survival. Patients’ serum concentrations were found to be dependent on the type of treatment used, including the timing of the debulking surgery (PDS vs. IDS). The prognostic value of HE4 concentration was found to be greater among patients who underwent primary debulking surgery.

## 6. Conclusions

The results of our study, together with the available literature, indicate an important role of HE4 in the diagnosis and treatment monitoring of ovarian cancer recurrence. Serum HE4 values may have prognostic potential. HE4 measurements at different treatment timepoints during second- and third-line chemotherapy treatment correlate with patient survival. There is a need for research in larger populations. Our study provides encouraging preliminary results showing a possible diagnostic and prognostic role of HE4 in recurrent ovarian cancer; further investigation on a larger study sample is required to confirm the obtained data. When compared to other screening methods including microRNA testing or diagnostic imaging methods, He4 seems to be a good and relatively inexpensive method for patient monitoring and diagnosis of OC relapse.

## Figures and Tables

**Table 1:** Patient characteristics.

Characteristics	Recurrence *n* = 96	No Recurrence *n* = 92
Age median (range)	60 (18–87)	62 (22–85)
Age (years)		
<60	44	34
≥60	52	58
FIGO stage, *n* of patients (%)		
I	3 (3.1%)	15 (16.3%)
II	4 (4.2%)	4 (4.4%)
III	86 (89.6%)	66 (71.7%)
IV	3 (3.1%)	7 (7.6%)
Histopathological classification, *n* (%)		
Serous	85 (88.5%)	75 (81.5%)
Non-serous	11 (11.5%)	17 (18.5%)
Grading		
1	4 (4.2%)	13 (14.1%)
2	17 (17.7%)	13 (14.1%)
3	75 (78.1%)	66 (71.8%)
Type *		
I	27 (28.1%)	25 (27.2%)
II	69 (71.9%)	67 (72.8%)
Type of surgery		
PDS (primary debulking surgery)	56 (58.3%)	59 (64.1%)
IDS (interval debulking surgery)	40 (41.7%)	33 (35.9%)
Residual disease		
0 (R0)	16 (16.7%)	28 (30.4%)
1 (<1 cm)	30 (31.3%)	27 (29.3%)
2 (>1 cm)	50 (52.0%)	37 (40.2%)
Intraperitoneal chemoteraphy *n* of patients = 24	10 (10.0%)	14 (15.0%)
Platinum sensitivity	62 (64.6%)	92 (100%)
Reccurence during the follow-up period		
No reccurence		92 (100%)
1 reccurence	56 (58%)	
>1 reccurence	40 (42%)	

* Type I OC: low grade serous (LGSC), mucinous (MOC), endometrioid (ENOC), clear cell carcinomas (CCOC) and malignant Brenner (transitional) tumors; Type II included high grade serous (HGSC), undifferentiated carcinomas and malignant mixed mesodermal tumors.

**Table 2:** Comparison of HE4 serum concentrations during the first line of treatment between the patients that developed and did not develop OC recurrence during the study period.

Characteristics	HE4 Concentration [pmol/L] During the First Line of Treatment Median (Range)	
	Recurrence *n* = 96	No Reccurence *n* = 92
HE4 at diagnosis	648.1 (37–7067)	295.5 (40.8–24,252)
	*p =* 0.015	
HE4 after IDS	142.7 (32.3–1234)	116.5 (40.9–2890)
	*p =* 0.677	
HE4 after PDS	129.1 (37.5–3786)	87.8 (33.7–6134)
	*p =* 0.009	
HE4 after the third chemotherapy during the first line of treatment	110.3 (21.3–1122)	77.3(33.9–222.4)
	*p =* 0.000	
HE4 after the 6th chemotherapy during the first line of treatment	85.9 (40.2–3060)	93.2 (39.3–661.5)
	*p =* 0.014	
HE4 at the end of first line of treatment	95.2 (33.4–3060)	79.2 (32.8–930.3)
	*p =* 0.294	

**Table 3:** Distribution of HE4 during at the time of OC recurrence diagnosis and treatment.

Prognostic Factor	HE4 Concentration [pmol/L] During the First Recurrence Median (Range)		
	At First Recurrence	After Third Treatment of Second-Line Chemotherapy	At the End of Second-Line Chemotherapy
Adjuvant	*n* = 56 185.1 (39.1–4909)	*n* = 53 143.1 (41.3–4560)	*n* = 49 129.4 (38.6–2788)
Neoadjuvant	*n* = 40 277.7 (30.7–3742)	*n* = 38 153.85 (37.0–3209)	*n* = 33 147.9 (36.0–16,367)
	*p =* 0.047	*p =* 0.237	*p =* 0.339
Low-grade	*n* = 21 192.1 (50.8–2182)	*n* = 20 131.1 (44.3–1161)	*n* = 19 133.5 (42.3–2154)
High-grade	*n* = 75 212.4 (30.7–4909)	*n* = 71 151.7 (37.0–4560)	*n* = 63 133.0 (36.0–16,367)
	*p =* 0.926	*p =* 0.598	*p =* 0.830
RT = 0 mm	*n* = 16 177.6 (30.7–3889)	*n* = 15 127.0 (37.0–4560)	*n* = 11 133.0 (36.0–1466)
RT > 0 ≤ 10 mm	*n* = 30 156.6 (39.1–1639)	*n* = 28 115.2 (41.3–1161)	*n* = 27 101.5 (38.6–2154)
	*p =* 0.344	*p =* 0.702	*p =* 0.530
RT = 0 mm	*n* = 16 177.6 (30.7–3889)	*n* = 15 127.0 (37.0–4560)	*n* = 11 133.0 (36.0–1466)
RT > 10 mm	*n* = 50 293.5 (50.8–4909)	*n* = 48 215.4 (45.3–3209)	*n* = 44 185.9 (47.4–16,367)
	*p* = 0.281	*p =* 0.146	*p =* 0.230
RT > 0 ≤ 10 mm	*n* = 30 156.6 (39.1–1639)	*n* = 28 115.2 (41.3–1161)	*n* = 27 101.5 (38.6–2154)
RT > 10 mm	*n* = 50 293.5 (50.8–4909)	*n* = 48 215.4 (45.3–3209)	*n* = 44 185.9 (47.4–16,367)
	*p =* 0.004	*p* = 0.006	*p =* 0.027
Serous	*n* = 85 212.4 (30.7–4909)	*n* = 80 150.1 (37.0–2560)	*n* = 72 133.2 (36–16,367)
Non-serous	*n* = 11 192.1 (58.1–2182)	*n* = 11 97.7 (61.6–864.4)	*n* = 10 134.2 (57.4–1466)
	*p =* 0.538	*p =* 0.697	*p =* 0.610
FIGO I and II	*n* = 7 159.1 (41.7–4909)	*n* = 7 143.1(41.3–2365)	*n* = 7 133.5(38.6–390.4)
FIGO III and IV	*n* = 89 212.4 (30.7–3889)	*n* = 84 150.15 (37.0–4560)	*n* = 75 133.0 (36.0–16,367)
	*p =* 0.678	*p =* 0.602	*p =* 0.613
Platinum-sensitive	*n* = 62 183.3 (30.7–4909)	*n* = 60 114.6 (37.0–2365)	*n* = 55 109.7 (36.0–16,367)
Platinum-resistant	*n* = 34 361.0 (58.1–3889)	*n* = 31 338.2 (49.5–4560)	*n* = 27 252.0 (66.9–2848)
	*p =* 0.024	*p <* 0.001	*p <* 0.001

RT—residual tumor tissue.

**Table 4:** HE4 serum concentration during the second OC reccurence.

Prognostic Factor	HE4 [pmol/L] during Second Recurrence Median (Range)		
	At Second Recurrence	After Third Treatment of Third Line Chemotherapy	At the End of Third Line Chemotherapy
Adjuvant	*n* = 22 220.0 (56.8–3150)	*n* = 19 139.0 (45.4–1496)	*n* = 18 148.7 (92.0–2887)
Neoadjuvant	*n* = 18 456.8 (43.1–3764)	*n* = 14 198.1 (26.7–1055)	*n* = 14 316.4 (33.3–2848)
	*p* = 0.242	*p* = 0.466	*p* = 0.447
Low–grade	*n* = 6 328.0 (89.7–1126)	*n* = 6 153.3 (85.1–603.2)	*n* = 6 229.4 (60.9–627.4)
High–grade	*n* = 34 276.9 (43.1–3764)	*n* = 27 151 (26.7–1496)	*n* = 26 184.7 (2.0–2887)
	*p* = 0.820	*p* = 0.889	*p* = 0.961
RT = 0 mm	*n* = 6 218.1 (42.1–746.7)	*n* = 6 147.1 (26.7–322.6)	*n* = 6 145.9 (33.3–2887)
RT > 0 ≤ 10 mm	*n* = 14 189.75 (56.8–1357)	*n* = 14 137.4 (45.4–1055)	*n* = 13 203.5 (2–1635)
	*p* = 0.804	*p* = 0.804	*p* = 0.861
RT = 0 mm	*n* = 6 218.1(42.1–746.7)	*n* = 6 147.1 (26.7–322.6)	*n* = 6 145.9 (33.3–2887)
RT > 10 mm	*n* = 20 682.6 (76.3–3764)	*n* = 13 180.7 (69.7–1496)	*n* = 13 311.3 (63.1–2848)
	*p* = 0.044	*p* = 0.219	*p* = 0.423
RT > 0 ≤ 10 mm	*n* = 14 189.7 (56.8–1357)	*n* = 14 137.4 (45.4–1055)	*n* = 13 203.5 (2.0–1635)
RT > 10 mm	*n* = 20 682.6 (76.3–3764)	*n* = 13 180.7 (69.7–1496)	*n* = 13 311.3 (63.1–2848)
	*p* = 0.012	*p* = 0.225	*p* = 0.248
Serous	*n* = 37 291.1 (43.1–3764)	*n* = 31 157.7 (26.7–1496)	*n* = 30 184.7 (2.0–2887)
Non–serous	*n* = 3 129.9 (108.1–233.0)	*n* = 2 136.9 (125.0–148.9)	*n* = 2 227.2 (131.2–323.2)
	*p* = 0.190	*p* = 0.546	*p* = 1.00
FIGO I and II	*n* = 3 1126 (140.9–2552)	*n* = 3 157.7 (92.1–1496)	*n* = 3 125.3 (85.6–736.2)
FIGO III and IV	*n* = 37 272.5 (43.1–3764)	*n* = 30 149.9 (26.7–1055)	*n* = 29 203.5 (2.0–2887)
	*p* = 0.248	*p* = 0.661	*p* = 0.771
Platinum sensitive	*n* = 29 291.1 (43.1–3764)	*n* = 25 151.0 (26.7–1496)	*n* = 23 203.5 (2.0–2887)
Platinum resistant	*n* = 11 241.4 (66.2–2122)	*n* = 8 149.6 (83.2–1001)	*n* = 9 166.0 (64.5–2848)
	*p* = 0.915	*p* = 0.866	*p* = 0.571

## Data Availability

The data presented in this study are available on request from the corresponding author. The data are not publicly available due to ethical restrictions.

## Appendix A. Assessment of HE4 Prognostic Values at Different Study Timepoints

**Table A1:** Assessment of serum HE4 prognostic value at the time of OC diagnosis and recurrence.

	AUC	Standard Error	Lower 95% CI	Upper 95% CI	*p*-Value
After primary surgical treatment and adjuvant chemotherapy					
2-year survival yes vs. no	0.78	0.07	0.64	0.92	*p <* 0.001
5-year survival yes vs. no	0.77	0.06	0.64	0.89	*p <* 0.001
After neoadjuvant chemotherapy and interval cytoreduction					
2-year survival yes vs. no	0.64	0.08	0.47	0.81	*p =* 0.101
5-year survival yes vs. no	0.56	0.09	0.37	0.74	*p =* 0.554
Platinum-sensitive patients					
2-year survival yes vs. no	0.79	0.07	0.64	0.93	*p <* 0.001
5-year survival yes vs. no	0.64	0.07	0.51	0.78	*p =* 0.036
Platinum-resistant patients					
2-year survival yes vs. no	0.59	0.10	0.39	0.79	*p =* 0.361
5-year survival yes vs. no	0.67	0.11	0.46	0.88	*p =* 0.011

**Table A2:** Assessment of serum HE4 prognostic value after the third course of second-line chemotherapy.

	AUC	Standard Error	Lower 95% CI	Upper 95% CI	*p*-Value
After primary surgical treatment and adjuvant chemotherapy					
2-year survival yes vs. no	0.75	0.08	0.59	0.90	*p =* 0.002
5-year survival yes vs. no	0.73	0.07	0.60	0.87	*p <* 0.001
After neoadjuvant chemotherapy and interval cytoreduction					
2-year survival yes vs. no	0.81	0.07	0.68	0.95	*p <* 0.001
5-year survival yes vs. no	0.63	0.09	0.46	0.81	*p =* 0.142
Platinum-sensitive patients					
2-year survival yes vs. no	0.81	0.09	0.63	0.10	*p <* 0.001
5-year survival yes vs. no	0.64	0.07	0.50	0.78	*p =* 0.057
Platinum-resistant patients					
2-year survival yes vs. no	0.56	0.11	0.35	0.77	*p =* 0.553
5-year survival yes vs. no	0.66	0.11	0.43	0.88	*p =* 0.169

**Table A3:** Assessment of serum HE4 prognostic value after the last course of second-line chemotherapy.

	AUC	Standard Error	Lower 95% CI	Upper 95% CI	*p*-Value
After primary surgical treatment and adjuvant chemotherapy					
2-year survival yes vs. no	0.75	0.09	0.58	0.92	*p =* 0.004
5-year survival yes vs. no	0.76	0.07	0.62	0.89	*p <* 0.001
After neoadjuvant chemotherapy and interval cytoreduction					
2-year survival yes vs. no	0.87	0.06	0.75	0.99	*p <* 0.001
5-year survival yes vs. no	0.71	0.09	0.52	0.89	*p =* 0.026
Platinum-sensitive patients					
2-year survival yes vs. no	0.83	0.05	0.73	0.93	*p <* 0.001
5-year survival yes vs. no	0.67	0.08	0.52	0.82	*p =* 0.03
Platinum-resistant patients					
2-year survival yes vs. no	0.60	0.12	0.37	0.84	*p =* 0.386
5-year survival yes vs. no	0.74	0.12	0.51	0.96	*p =* 0.043

**Table A4:** Assessment of serum HE4 prognostic value at the time of diagnosis of II relapse.

	AUC	Standard Error	Lower 95% CI	Upper 95% CI	*p*-Value
After primary surgical treatment and adjuvant chemotherapy					
2-year survival yes vs. no	1.00	0.00	1.00	1.00	*p* = 0.00
5-year survival yes vs. no	0.82	0.09	0.68	0.10	*p <* 0.001
After neoadjuvant chemotherapy and interval cytoreduction					
2-year survival yes vs. no	0.35	0.14	0.08	0.62	*p =* 0.271
5-year survival yes vs. no	0.65	0.14	0.38	0.92	*p =* 0.272
Platinum-sensitive patients					
2-year survival yes vs. no	0.96	0.03	0.90	1.00	*p* < 0.001
5-year survival yes vs. no	0.71	0.10	0.53	0.90	*p =* 0.025
Platinum-resistant patients					
2-year survival yes vs. no	0.70	0.14	0.42	0.98	*p =* 0.519
5-year survival yes vs. no	0.92	0.09	0.74	1.00	*p* < 0.001

**Table A5:** Assessment of serum HE4 prognostic value after the third course of third-line chemotherapy treatment.

	AUC	Standard Error	Lower 95% CI	Upper 95% CI	*p*-Value
After primary surgical treatment and adjuvant chemotherapy					
2-year survival yes vs. no	0.33	0.11	0.12	0.55	*p =* 0.134
5-year survival yes vs. no	0.73	0.12	0.50	0.96	*p =* 0.048
After neoadjuvant chemotherapy and interval cytoreduction					
2-year survival yes vs. no	0.49	0.18	0.14	0.84	*p =* 0.954
5-year survival yes vs. no	0.51	0.18	0.16	0.86	*p =* 0.954
Platinum-sensitive patients					
2-year survival yes vs. no	0.36	0.119	0.14	0.61	*p =* 0.300
5-year survival yes vs. no	0.62	0.119	0.39	0.86	*p =* 0.300
Platinum-resistant patients					
2-year survival yes vs. no	0.29	0.17	−0.05	0.62	*p =* 0.209
5-year survival yes vs. no	0.73	0.19	0.35	1.00	*p =* 0.231

**Table A6:** Assessment of serum HE4 prognostic value after the last course of third-line chemotherapy.

	AUC	Standard Error	Lower 95% CI	Upper 95% CI	*p*-Value
After primary surgical treatment and adjuvant chemotherapy					
2-year survival yes vs. no	0.23	0.10	0.03	0.44	*p =* 0.010
5-year survival yes vs. no	0.80	0.11	0.58	1.00	*p* = 0.008
After neoadjuvant chemotherapy and interval cytoreduction					
2-year survival yes vs. no	0.41	0.17	0.08	0.74	*p =* 0.586
5-year survival yes vs. no	0.59	0.17	0.26	0.92	*p =* 0.586
Platinum-sensitive patients					
2-year survival yes vs. no	0.29	0.11	0.07	0.51	*p =* 0.067
5-year survival yes vs. no	0.71	0.11	0.48	0.93	*p =* 0.067
Platinum-resistant patients					
2-year survival yes vs. no	0.25	0.15	−0.05	0.55	*p =* 0.102
5-year survival yes vs. no	0.72	0.20	0.33	1.00	*p =* 0.262
